# The Roles of Histone Lysine Methyltransferases in Heart Development and Disease

**DOI:** 10.3390/jcdd10070305

**Published:** 2023-07-18

**Authors:** Jun-yi Zhu, Joyce van de Leemput, Zhe Han

**Affiliations:** 1 Center for Precision Disease Modeling, Department of Medicine, University of Maryland School of Medicine, Baltimore, MD 21201, USA; 2 Division of Endocrinology, Diabetes, and Nutrition, Department of Medicine, University of Maryland School of Medicine, Baltimore, MD 21201, USA

**Keywords:** histone lysine methyltransferase, cardiac development, congenital heart disease, epigenetic regulation

## Abstract

Epigenetic marks regulate the transcriptomic landscape by facilitating the structural packing and unwinding of the genome, which is tightly folded inside the nucleus. Lysine-specific histone methylation is one such mark. It plays crucial roles during development, including in cell fate decisions, in tissue patterning, and in regulating cellular metabolic processes. It has also been associated with varying human developmental disorders. Heart disease has been linked to deregulated histone lysine methylation, and lysine-specific methyltransferases (KMTs) are overrepresented, i.e., more numerous than expected by chance, among the genes with variants associated with congenital heart disease. This review outlines the available evidence to support a role for individual KMTs in heart development and/or disease, including genetic associations in patients and supporting cell culture and animal model studies. It concludes with new advances in the field and new opportunities for treatment.

## 1. Introduction

Our genome is stored tightly wrapped around histone proteins as chromatin. This compaction is necessary to fit inside the cell’s nucleus. Inherently, this necessitates a system to ensure the genome remains accessible for gene expression [1]. For this purpose, epigenetic regulation leaves a trail of marks that facilitate both the structural packing and unwinding of the genome. Histones are largely globular structures organized into octameric nucleosomes (four dimers defined by H3-H4 and H2A-H2B histone pairs), with unstructured tails [2]. Histone modification marks on the tails can take on different forms, of which methylation, at lysines or arginines, and acetylation are the most common forms that regulate gene expression [3]. Methylation stands out for its specificity—lysine-specific methyltransferases (KMTs) target a single lysine (K) on a single histone (H)—and flexibility, with methylation marks both activating and repressing transcription, making it one of the principal chromatin regulatory mechanisms acting upon fundamental nuclear processes [4].

**Figure 1:**
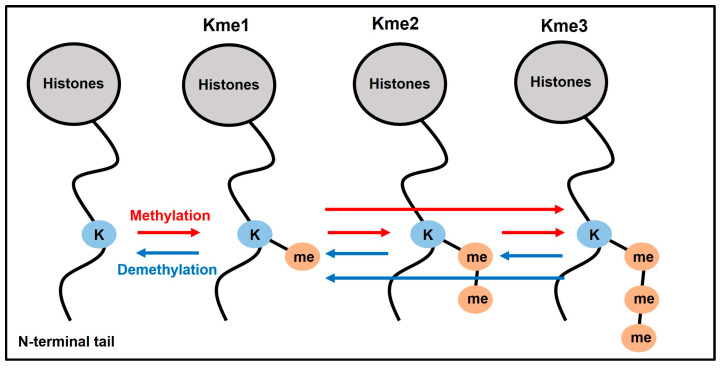
**Dynamics of histone lysine methylation and demethylation.** The methylation marks regulate structural unwinding and packing of the genome, which facilitates and represses gene expression, respectively. Methylases and demethylases combined offer a dynamic regulatory system that can respond to endogenous and environmental changes. The enzymes catalyze addition and removal of methylation marks, either stepwise (single) or multiple marks at once. K, lysine; me, methyl group (CH_3_).

Methylation of lysine residues at the N-terminal tails of histone H3 and the lysine at the H4 N-terminus regulate major genomic functional processes [5]. Simply put, three of the lysine methylation sites have been implicated in transcriptional activation: H3K4, H3K36, and H3K79 (H3K4 and H3K36 have been associated with transcriptional initiation and elongation, respectively); and three other sites have been implicated in transcriptional repression: H3K9, H3K27, and H4K20 [6]. Each lysine residue can accept up to three methyl groups to form mono-, di-, and trimethylated derivatives (Kme1, Kme2, and Kme3, respectively) (Figure 1). Further regulatory finetuning is achieved by balancing the activity of KMT with that of lysine demethylases (KDMs) (Figure 1). This shifts the epigenetic regulatory landscape from static to dynamic. Different methylation sites can act together to ‘‘poise’’ genes, which enables rapid activation upon lineage commitment. These double-methylated, i.e., bivalent, promoters carry marks for both activation and repression, so that the definitive cell types carry marks either in support of a transcriptionally active or a silent state, thus sustaining cell fate decisions [7]. Over fifty proteins encoded in the human genome are known or predicted KMTs (catalytic SET domain KMTs) [8]; each targets a specific histone-lysine (H_K_) and catalyzes any or a combination of me1, me2, or me3 [5]. In addition, over thirty histone demethylases (KDMs) have been described in humans, which can be broadly divided into lysine-specific demethylases (LSD, including nine KDM subfamilies) and the Jumonji C (JmjC) histone demethylases [9,10]. Some methylation marks are preferentially enriched at promoters of regulatory genes that control a variety of developmental pathways. The surrounding positioning sequence, spacing, and combination of methylation marks provide the context that links the methylated sites to downstream biological functions mediated by methyl lysine-binding proteins [2].

During development, enormous transcriptional shifts take place. Methylation plays a crucial role to guide these transitions. In line with this are findings of deregulated methylation marks in human aging and developmental disorders, as well as cancer [5,11,12,13]. Likewise, histone lysine methylation has been shown to be important during heart development, and its deregulation has been linked to cardiac disease [14,15,16]. Similarly, KDMs show preferential expression during fetal and adult stages of heart development [16]. Studies using animal models have demonstrated their importance for heart development [17,18,19,20], and several KDMs have shown differential expression in the heart of patients with heart failure [21,22,23]. Moreover, deleterious de novo mutations in *KDM5A* and *KDM5B* have been associated with congenital heart disease [24,25].

**Figure 2:**
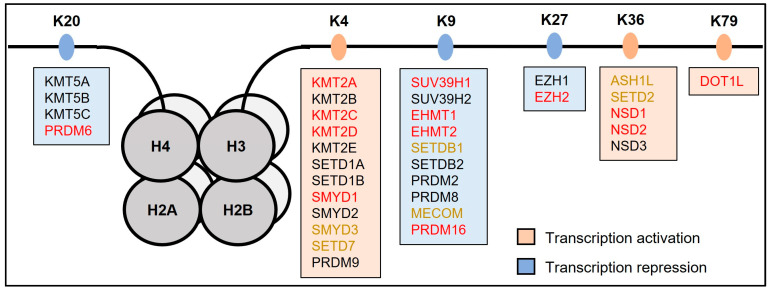
**Histone lysine-specific methyltransferases (KMTs) and their targets.** Each grey ball represents a globular histone (H), together organized into a nucleosome (an octamer consisting of two identical subunits of the core histones H4, H3, H2A, and H2B). The black line represents the unstructured N-terminal ends of the H4 an H3 histones that contain the lysine (K) methylation sites. Red font, KMT with sufficient evidence to support a regulatory role during heart development and/or disease (defined by supporting data from multiple model systems, which can be strengthened by disease association); yellow font, limited suggestive evidence (defined by supporting data from a single model system); and black font, currently no data to indicate involvement. Details have been described in this review.

Recently, Davies et al. wrote a comprehensive review on demethylases in the heart [16]. Considering the growing number of genetic associations between KMTs and heart disease within the past decade, herein, we outline the currently available evidence, both in patients (genetic association studies) and supporting in vitro and in vivo (including animal) models, to support the role of KMTs in heart development and/or disease (Figure 2). We conclude with next steps in moving the field forward and how a greater understanding of the epigenetic contributions might open new avenues for treatment to ultimately benefit the patients.

## 2. Lysine-Specific Methylation in Heart Development and Disease

The SET domain lysine methyltransferases are active KMTs, and their SET domain catalyzes methylation of specific lysine marks at both histone and non-histone substrates [4]. The domain was first discovered in *Drosophila*, where three proteins shared the conserved SET sequence: the modifier of position-effect variegation, Suppressor of variegation 3-9 [Su(var)3-9]; the polycomb-group chromatin regulator, Enhancer of zeste [E(z)]; and the trithorax-group chromatin regulator, Trithorax (Trx) [4,26]. Proteins carrying variants of the approximately 130-amino-acid SET domain have been identified in the genomes of all eukaryotic organisms sequenced to date, as well as many bacterial genomes [26,27,28].

Below, the KMTs have been grouped by the histone-lysine (H_K_) mark at which they are primarily active. For each methyltransferase, the available evidence from genetic association studies in patients that have linked variants in that KMT to heart disease (Table 1) and for its role in heart development, including evidence from cell and animal model studies, have been provided (Figure 2).

### 2.1. H3K4 Methylation

**KMT2A** (HUGO Gene Nomenclature Committee, HGNC, alias: MLL1, MLL1A, TRX1) is a histone lysine methyltransferase that belongs to the SET1 family, members of which have the SET domain, which is responsible for their histone methyltransferase activity. Mutations in *KMT2A* cause Wiedemann−Steiner syndrome (WSS; OMIM #605130), a rare congenital disease characterized by excessive hair growth, short stature, and distinctive facial features [29,30]. Early on, a subpopulation of patients was misdiagnosed with Kabuki syndrome (KS; OMIM #147920), another multiple congenital anomaly syndrome that commonly presents with heart disease, which is caused by variants in *KMT2D* [30]. Since then, the phenotypic spectrum of Wiedemann−Steiner syndrome has been expanded to include congenital heart disease and other phenotypes. In fact, one study reported cardiac abnormalities, mostly of a structural nature, in ~35% of patients [31].

In *Drosophila*, silencing *trx*, the fly homolog of mammalian *KMT2A* and *KMT2B* in the heart, led to non-detectable cardiac actin indicative of a completely absent heart tube, as well as a reduced lifespan [32]. These findings in the fly support the causative role of *KMT2A* variants in congenital heart disease. 

**KMT2D** (HGNC alias: MLL4) is another member of the SET1 family, and like KMT2A, acts as a histone lysine methyltransferase. Variants in *KMT2D* cause Kabuki syndrome, a rare congenital disease marked by multiple anomalies, including short stature, distinctive facial features, intellectual disability, persistent fingertip pads, and skeletal abnormalities. Kabuki syndrome frequently presents with congenital heart defects (~70% of patients), which display a unique predilection for left-sided obstructive lesions [33,34,35]. Additional variants in *KMT2D* have been reported in patients with varying cardiac diseases, including left-sided cardiac lesions, heterotaxy, left heart hypoplasia, and a multiple congenital anomaly syndrome distinct from Kabuki syndrome [36,37,38,39,40].

Multiple animal models have been used to study the role and mechanism of KMT2D in heart development and disease. A study in mice demonstrated the importance of KMT2D in cardiac precursors and cardiomyocytes during heart development [34]. It showed that deletion of *Kmt2d* in the myocardium results in decreased H3K4me1 and H3K4me2 levels at enhancers and promoters, which affects the expression of genes involved in ion homeostasis/transport (*Atp1a2*, *Fxyd1*, *Atp2b4*, *Snta1*, and *Kcnj12*), wounding/immune/inflammatory response (*Tgfb1*, *Ccl2*, and *Ccl7*), hypoxia–reoxygenation (*Vegfa*, *Hmox1*, and *Gpx3*), and cell cycle regulation/differentiation (*Eid1*, *Smyd1*, *Nkx2-5*, *Snta1*, and *Prrx2*) [34]. A frog (*Xenopus laevis*) *kmt2d* knockdown model showed hypoplastic hearts that lacked the three-chambered structure due to abnormal development of the first and second heart fields and abnormal cardiac differentiation [41]. This model mimics the hypoplastic heart defects observed in patients with Kabuki syndrome. Zebrafish (*Danio rerio*) *kmt2d* null mutants displayed a complex phenotype that resembles the foremost features of Kabuki syndrome, including microcephaly, palate defects, abnormal ear development, and cardiac defects [42]. The cardiac phenotype was related to a vasculogenesis defect that affected endocardium patterning and, as a result, the formation of the heart ventricle lumen, as well as angiogenesis defects. They further found hyperactive *Notch* signaling in endocardial and endothelial cells of the *kmt2d* null zebrafish, which was associated with increased Rbpj protein levels, a Notch transcription factor [42]. These findings encourage clinical pursuit in patients with Kabuki syndrome to determine whether the role of KMT2D−Notch signaling in heart development is conserved. In *Drosophila*, the *Lpt* and *trr* genes encode the N-terminal and C-terminal homologs, respectively, of mammalian KMT2C and KMT2D [43]. Silencing either *Lpt* or *trr,* or silencing them simultaneously, in the fly heart led to similar abnormal cardiac morphology, tissue fibrosis, and cardiac functional defects. Like *KMT2D*, *Lpt,* and *trr* were found to modulate histone H3K4me1 and H3K4me2 [43]; and, homologs of the differentially expressed genes in Kmtd-targeted genes in mice were affected in the *Lpt*- and *trr*-deficient flies, including genes involved in ion transport/binding (*Ca-α1T* [mouse *Cacna1h*], *CaMKII* [mouse *CamK2a*], *Syn* [mouse *Snta1*], and *Ype* [mouse *Ypel5*]), hypoxia (*CG15116* [mouse *Gpx1*] and *CG3156* [mouse *Abcb10*]), cell cycle regulation (*E2f1* [mouse *E2f2*]), and more [43]. Altogether, these animal studies support a conserved role for KMT2D in heart development and provide insight into the underlying molecular–genetic mechanisms.

**KMT2C** (HGNC alias: MLL3) is another histone lysine methyltransferase belonging to the SET1 family. Both mRNA and protein levels of KMT2C were increased in the left ventricles of hearts from patients with dilated cardiomyopathy (DCM) that underwent heart transplantation [44]. These expression levels correlated with pathological severity, and accompanying increased H3K4me2, but not H3K4me3, marks were observed in the hearts of patients [44]. Moreover, they found increased protein expression for Smad3, GATA4, and EGR1 in hearts with dilated cardiomyopathy. All three are key mediators of cardiomyocyte hypertrophy and cardiac fibrosis, which are prominent features of dilated cardiomyopathy [44]. In another study, exome sequences revealed likely causal de novo variants in *KMT2C* and *KDM5A,* both histone methylases, that segregated with disease in a family with a history of congenital heart disease [45]. However, a direct link from KMT2C to heart development or congenital heart disease has not yet been shown.

A mouse model of cardiac remodeling (based on transverse aortic constriction) showed a significant increase in mRNA levels for *KMT2C* [44]. Like the patients with dilated cardiomyopathy, the mice displayed increased left ventricular end diastolic and systolic dimensions, and reduced fractional shortening, as well as evidence of collagen deposition [44]. These findings suggest a conserved role for KMT2C in the pathomechanism underlying dilated cardiomyopathy. Notable in the mouse hearts, unlike the human hearts, mRNA levels for *SETD1A* were decreased, which might be due to the pressure-induced, rather than genetic-induced, disease causation in this model [44].

**SETD7** (HGNC alias: KMT7, SET7, SET7/9, Set9) is a histone methyltransferase that specifically regulates H3K4 monomethylation (H3K4me1) [46]. SETD7 is highly expressed in the developing heart of zebrafish (*Danio rerio*). Knockdown of *setd7* (antisense morpholino) during this time did not change the expression pattern of heart markers; however, it did lead to severe defects in the zebrafish heart marked by cardiac edema (i.e., swelling due to decreased blood flow) [47]. Moreover, simultaneous knockdown of *setd7* and *smyd3*, another H3K4 methyltransferase, during development caused synergistic cardiac defects [47]. Using in vitro human embryonic stem cell- and human induced pluripotent stem cell (iPSC)-derived cardiomyocytes, it was shown that SETD7 is a key regulator of cardiac lineage commitment [48]. It does so by interaction with key co-factors at distinct stages of differentiation. Notably, besides methylation of H3K4me1, SETD7 was shown to mediate target gene expression via association with H3K36me3 modification on those target genes, which included essential components of the SWI/SNF chromatin-remodeling complex and *NKX2-5*, one of the major cardiac transcription factors [48]. SETD7 is also required for calcium processes in terminally differentiated cardiomyocytes, in which calcium signaling is key for contraction and, thus, regulating the heart beat [48].

**SMYD3** (HGNC alias: KMT3E), a SMYD family protein, is expressed ubiquitously during development in zebrafish (*Danio rerio*) [49,50]. Aside from the synergistic action with SETD7 during heart development [47], both deficiency (antisense morpholino) and overexpression of *smyd3* by itself during zebrafish development causes heart defects [47]. These cardiac defects are marked by pericardial edema and the abnormal expression of the known heart-chamber markers *cmlc2*, *amhc,* and *vmhc*, as well as the abnormal expression of the myogenic regulatory factors *myod* and *myog* [50]. These findings support an important role for SMYD3 in heart development.

**SMYD1** (HGNC alias: KMT3D, BOP) is a striated muscle-specific H3K4 methyltransferase, unlike the ubiquitously expressed SMYD3. The expression of SMYD1 (then named BOP) was significantly increased in the explanted heart of patients with end-stage heart failure, which correlated with reduced expression of cardiac genes [51]. Later, variants in BOP (SMYD1) were identified in patients diagnosed with hypertrophic cardiomyopathy [52]. However, these studies did not yet determine the molecular–genetic role of SMYD1 during heart development.

An early study using chicks and mice found that BOP acts as a histone deacetylase-dependent transcriptional repressor and was expressed in cardiac and skeletal muscle precursors prior to differentiation [53]. Moreover, knockdown of *BOP* in mice disrupted the maturation of ventricular cardiomyocytes and impeded development of the right ventricle. The expression of *Hand2*, an established and essential component for right ventricular development, was dependent on BOP [53]. In zebrafish, it was shown that of the two SmyD1 forms through alternative splicing, loss of *smyd1b* induced cardiac muscle defects; these were more severe in double (*smyd1a*, *smyd1b*) mutants (antisense morpholino) [54,55,56]. The cardiac defects were attributed to aberrant sarcomere organization, which was accompanied by increased expression of *hsp40*, *hsp90a1*, and possibly *unc45b* [55,56]. In mice, Smyd1 has been shown to be essential for cardiomyocyte differentiation and cardiac morphogenesis [53]. Conditional knockdown of *Smyd1* in either the first or second heart field, specifically in mice, demonstrated its crucial role in maintaining cardiomyocyte proliferation during at least two distinct embryonic heart developmental stages. Upon loss of Smyd1 in the heart, oxidative and endoplasmic reticulum (ER) stress responses induced early lethality [57]. The role of Smyd1 in healthy early heart development is mediated through its interaction with ASHL2, which activates the *Isl1* promoter by H3K4me3, thereby increasing *Isl1* expression. In addition, Smyd1 interacts with HDAC via the deacetylase inhibitor TSA, which leads to decreased expression of *ANF* [58], a cardiac natriuretic peptide and established marker for heart failure.

In adult mice, the importance of SMYD1 in restricting heart growth has been shown. Smyd1 was upregulated in a mouse model of hypertrophy and heart failure, and loss of function resulted in cellular hypertrophy and remodeling, ultimately leading to heart failure [59,60]. Smyd1 regulated the expression of genes associated with cardiac pathology, including mitochondrial energetics and cardiac energetics [59,60]. 

To date, no studies have shown association with heart development for any of the other H3K4 methylases, such as KMT2B (HGNC alias: MLL2, TRX2, MLL1B, MLL4), KMT2E (HGNC alias: SETD5B), SETD1A, SETD1B (HGNC alias: KMT2G), and PRDM9 (HGNC alias: KMT8B) [61]. Notably, cardiomyocytes-specific deletion of *Smyd2* (SMYD2; HGNC alias: KMT3C) showed it was dispensable for heart structural and functional development in mice, and it had no effect on H3K4 or H3K36 methylation [62].

### 2.2. H3K9 Methylation

**SUV39H1** (HGNC alias: KMT1A) and **SUV39H2** (HGNC alias: KMT1B) are members of the SET domain-containing histone lysine methyltransferase family and have similar enzymatic activities. Each regulates H3K9 methylation, which represses gene transcription. Histone methylation (H3K4me3, H3K9me2, and H3K9me3) was reduced in the left ventricles of patients diagnosed with end-stage non-ischemic dilated cardiomyopathy [63]. Upon intervention through implantation of a left ventricular assist device (LVAD), the methylation levels were restored, with concurrent upregulation of SUV39H1 [63]. In another study, MCP1 (HGNC alias: CCL2) was found significantly upregulated in CD14+ monocytes of patients with coronary heart disease. These cardiac lesions have been linked to an inflammatory response. In the patient CD14+ monocytes, levels of SUV39H1, regulatory factor (RFX1), and histone deacetylase (HDAC1) were reduced at the *MCP1* promoter region [64]. These studies indirectly implicate SUV39H1 in cardiac disease.

Studies in mice and cell lines similarly, yet indirectly, suggest a role for SUV39H1 in cardiac disease. Kindlin-2 is an important regulator of cardiac structure and function. It interacts with SUV39H1 to facilitate recruitment of this KMT to the *GATA4* promoter to catalyze H3K9me2 and H3K9me3 methylation [65]. GATA4 is an established key transcription factor during cardiogenesis, and disruption of this system in mice led to hypertrophic cardiomyopathy [65]. An in vitro study using C2C12 mouse myoblasts and mouse embryonic fibroblasts showed the importance of Suv39h1-mediated H3K9 methylation on the promoters of myogenic differentiation genes. Repression of gene transcription to activate muscle differentiation was removed by SETD7 (HGNC alias: SET7) [66]. Whether this is also true in vivo and in cardiac muscle remains to be determined.

While these studies provide some evidence to implicate SUV39H1 in cardiac diseases, currently there is no evidence to support or deny a similar role for SUV39H2.

**EHMT1** (HGNC alias: KMT1D) and **EHMT2** (HGNC alias: KMT1C, G9A) are also members of the SET domain-containing histone lysine methyltransferase family, and both have similar enzymatic activities that regulate H3K9 methylation to repress gene transcription. Variants in *EHMT1*, like those in *KMT2C*, have been found causative in Kleefstra syndrome [67]. Of the patients with Kleefstra syndrome attributed to mutations in *EHMT1*, ~40–45% have cardiac defects; even in the absence of structural heart defects, cardiac arrhythmia has been observed in these patients [68].

In vitro and animal model studies for EHMT1 and its variants have often focused on the neurodevelopmental aspects of Kleefstra syndrome; thus, many questions remain about the pathomechanism by which EHMT1 might contribute to congenital heart disease. For EHMT2, knockdown of *G9A* (an alias) significantly reduced H3K9me2 methylation in rat bone mesenchymal stem cell-derived cardiomyocytes [69]. This coincided with increased expression of several transcription factors (GATA4, NKX2-5, and MEF2C) known to be important during cardiomyocyte formation during early-stage myocardial development [69]. A knockdown study in mice demonstrated the importance of G9A in preventing hypertrophy in the adult heart. G9A interaction with EZH2, the catalytic subunit of polycomb repressive complex 2 (PRC2), regulated its H3K27me3 activity, and interaction with MEF2C maintained the heterochromatin structure that is required to silence developmental genes in the adult cardiomyocytes [70]. A G9A-specific inhibitor, BIX01294, was able to expand mouse cardiac progenitor cells without affecting their phenotype nor their potential to differentiate [71]. Altogether, this demonstrates the importance of EHMT2 in regulating cardiomyocyte hypertrophy.

**SETDB1** (HGNC alias: KMT1E) is an H3K9 methyltransferase. Jarid2 is a key regulator of cardiovascular development, as well as numerous other developmental processes. It requires SETDB1 methylation activity to repress the expression of target genes, including *Notch1*, during heart development in mice [72]. However, aside from this study, it remains unclear to what extent SETDB1 is involved in heart development and cardiac disease.

**MECOM** (HGNC alias: KMT8E, PRDM3) displays histone H3K9 methyltransferase activity and was initially described as an oncogenic putative transcription factor. Targeted mutagenesis to disrupt the full-length *Evi1* (mouse homolog of MECOM) in mouse embryonic stem cells demonstrated its importance for many developmental processes, including heart development [73]. Mice deficient in *Evi1* exhibited severe congenital heart defects that resulted in perinatal death [74]. Many genes that had been previously associated with congenital heart disease were found to carry a known Evi1-binding site, and expression of 18 of these genes was found dysregulated by *Evi1* siRNA knockdown [74]. These findings suggest MECOM could play a central role in transcriptional regulation during cardiac development.

**PRDM16** (HGNC alias: KMT8F) is a transcription factor that mediates H3K9 methylation. PRDM16 is localized in the nuclei of cardiomyocytes, throughout development (in mice and humans) and in the adult heart [75]. Variants of *PRDM16* have been associated with non-syndromic left ventricular noncompaction cardiomyopathy (LVNC), dilated cardiomyopathy associated with 1p36 deletion syndrome [75,76], and pediatric dilated cardiomyopathy [77].

Zebrafish with PRDM16 haploinsufficiency or a human truncation mutant showed contractive dysfunction and partial uncoupling of cardiomyocytes, as well as impaired cardiomyocyte proliferative capacity [75]. *Prdm16* was dispensable for early heart development in mice; however, deficiency led to later-stage cardiac hypertrophy, adverse remodeling, and ultimately heart failure. The hypertrophic genes were regulated by Prdm16, together with Ehmt1/2 [78]. Furthermore, in the adult heart, Pdrm16 played a crucial role in maintaining mitochondrial function and preventing metabolic stress [78]. The hearts of mice with cardiac-specific *Prdm16* conditional knockout showed abnormal cardiac conduction and phenotypes associated with cardiomyopathy, including cardiac fibrosis and hypertrophy [79]. These were accompanied by impaired ion homeostasis (Ca^2+^, K^+^, and Na^+^) in left ventricular heart tissue [79]. In addition, cardiomyocyte-specific *Prdm16* knockout mice displayed LVNC reminiscent of that seen in patients with variants in *PRDM16* [80]. Prdm16 transcription factor activity was enriched in the left ventricular compacted myocardium in these mice, where it activated compact myocardial genes (*Hey2* and *Mb*), while repressing trabecular myocardial (*Nppa*, *Cited1*, *Nppb*, and *Mest*) and neural (*Cttnbp2* and *Spon1*) genes, thus maintaining their transcriptional identity [80].

***PRDM2*** (HGNC alias: *KMT8A*) is near a candidate susceptibility locus for cardiotoxicity (characterized by progressive systolic dysfunction of the left ventricle) induced by the chemotherapeutic agent anthracycline, as identified by genome-wide sequence association in a human cohort [81], providing a first hint at its possible role in heart development and/or disease.

No studies have yet provided evidence to support a role for other H3K9 methylases, such as SETDB2 (HGNC alias: KMT1F), and PRDM8 (HGNC alias: KMT8D) in heart development or disease.

### 2.3. H3K27 Methylation

**EZH1** (HGNC alias: KMT6B) and **EZH2** (HGNC alias: KMT6A) are alternative catalytic components of the polycomb repressive complex 2 (PRC2), which is highly conserved from *Drosophila* to primates [82]. It has been tasked with H3K27 mono-, di-, and trimethylation (H3K27me1/2/3), which enacts gene transcription repression during cell development, differentiation, and cell fate [82].

EZH2 upregulation has been described in varying cardiac diseases, including in heart tissue from patients with end-stage dilated cardiomyopathy [83], in atrial muscle and atrial fibroblasts from patients with atrial fibrillation (marked by significant atrial fibrosis and atrial fibroblast differentiation) [84], and in serum from patients with coronary heart disease [85]. Both EZH2 and H3K27me3 were increased in heart tissue from patients with ischemic heart disease [86].

The EZH1 and EZH2 catalytic domains of PRC2 show partial functional redundancy. However, growing evidence suggests functional specialization, with EZH1 dominant during heart regeneration and EZH2 dominant during heart development. In fact, *Ezh1* knockdown by itself did not affect heart development or function [87]. However, Ezh1, but not Ezh2, was required for regeneration following myocardial infarction, i.e., injury, in mice. PRC2 composition is different for each: where Ezh1 acts with the suppressor of the Zeste 12 (Suz12) component [87], Ezh2 prefers the embryonic ectoderm development (Eed) component [88]. The findings indicate that a different epigenetic response is required for development and regeneration of the heart [87]. This is also reflected in their gene expression profiles: Ezh1 was shown to upregulate genes associated with cardiac muscle growth in mice [87], whereas Ezh2 repressed mesenchymal and neuronal gene programs in mouse cardiomyocytes [88]. Furthermore, heart-specific knockdown of *Ezh2* in mice resulted in decreased cardiomyocyte proliferation, increased apoptosis, and dysregulated endothelial-to-mesenchymal transition. This was accompanied by varied cardiovascular malformations, such as hypoplastic endocardial cushions, which led to perinatal death, and were linked to the downstream target Hey2 [89]. Hey2 plays a key role during heart development [90,91], and genetic variants have been associated with congenital heart disease [92]. Conditional knockout of *Ezh2* in cardiomyocytes halted mouse neonatal heart regeneration due to impeded proliferation of cardiomyocytes and H3K27me3 modifications. This was linked to PDGFR-β signaling [93]. Like with patients, mouse models of atrial fibrosis showed increased EZH2, and both pharmacological (GSK126; a selective EZH2 inhibitor) and molecular inhibition of EZH2 attenuated the cardiac phenotype. The underlying pathomechanism involved EZH2-mediated Smad signaling to promote fibroblast differentiation [84]. Disrupted interaction of EZH2 with Smad has also been associated with cardiac fibrosis. Long noncoding RNA NEAT1 is upregulated in patients with heart failure. A mouse model showed this results in increased recruitment of Ezh2 to the promotor region of Smad7, thus repressing its expression and promoting cardiac fibrosis [94]. Heart tissue from mice with myocardial infarction, like tissue from patients with ischemic heart disease, showed increased EZH2 and H3K27me3, as well as reduced Na+ channel (Nav1.5) expression [86]. Again, both pharmacological (GSK126) and molecular inhibition of EZH2 attenuated the cardiac phenotype [86]. *Ezh2*-deficient cardiac progenitors in mice showed postnatal myocardial pathology and disrupted cardiac gene expression, including increased *Six1*, which induced cardiomyocyte hypertrophy and increased the expression of skeletal muscle genes [95]. Together, these animal model studies, like the patient studies, demonstrate the diversity of cardiac conditions in which EZH2 plays a role. Given the partial functional redundancy between EZH1 and EZH2, additional studies into the role of EZH1 in these cardiac disease models is warranted, especially as a potential therapeutic target to offer protection following cardiac injury [87].

### 2.4. H3K36 Methylation 

**ASH1L** (HGNC alias: KMT2H) is a histone lysine methyltransferase with H3K36-specific (H3K36me1 and -me2) activity, which antagonizes polycomb silencing, thus acting as a transcriptional activator [96,97,98]. Genetic variants in *ASH1L* have been associated with congenital heart disease [25,99,100]

Preliminary data in *Drosophila* suggest that Ash1 (fly homolog of ASH1L) plays a role in heart development (*manuscript in preparation*).

**SETD2** (HGNC alias: HIF-1, KMT3A) is an H3K36me3 histone-lysine N-methyltransferase [101]. *Setd2* is highly expressed across embryonic stages in mice and is largely responsible for H3K36me3 in the mouse heart. Indeed, cardiac progenitors deficient for *Setd2* showed notable coronary vascular defects and ventricular non-compaction, which resulted in fetus lethality at mid-gestation in mice [102]. These mice showed greatly decreased H3K36me3, associated with dysregulated expression of key cardiac genes, including *Rspo3* and *Flrt2* [102]. The findings support a role for SETD2 in cardiovascular development and, by extension, disease.

**NSD1** (HGNC alias: KMT3B) and **NSD2** (HGNC alias: KMT3G; MMSET) both enact H3K36me1 and H3K36me2 activity [103,104,105,106]. Mutations in NSD1 have been identified as the genetic cause of Sotos syndrome, which is characterized by pre- and postnatal overgrowth, notable craniofacial features, advanced bone age, and developmental delay. Moreover, a subset of patients presents with congenital heart defects [107,108,109]. A de novo variant in *NSD1* was associated with atrioventricular septal defect, a specific cardiac malformation that presented as a congenital heart disease [110]; and, *NSD1* genetic variants have been associated with congenital heart disease [25,99]. *NSD2* (HGNC previous symbol: WHSC1) lies in the critical deletion region of Wolf–Hirschhorn syndrome (WHS; OMIM #194190), a neurodevelopmental disorder marked by facial dysmorphology and growth retardation, of which a subset of patients presented with congenital heart disease [111]. Finally, variants in *NSD2* have been associated with congenital heart disease [100]; in fact, both loss-of-function (LoF) and gain-of-function (GoF) variants in *NSD2* have been associated with abnormal heart morphology linked to disrupted cardiac patterning [100,112].

The molecular mechanism of NSD1 in heart development is unknown. Mice deficient in *Nsd2* showed delayed growth, midline defects, and related congenital cardiovascular defects reminiscent of Wolf–Hirschhorn syndrome [113]. *Nsd2* genetically interacted with the *Nkx2-5* transcription factor, which disrupted gene expression in the developing embryonic mouse hearts [113]. In addition, a mouse model of cardiac hypertrophy with concurrent conditional knockdown of *Nsd2* in the myocardium resulted in a milder ventricular remodeling phenotype and improved cardiac function, attributed to reduced H3K36me2 [114], thus demonstrating a role for NSD2 in ventricular remodeling.

A role for NSD3 (HGNC alias: KMT3F)-mediated H3K36 methylation during heart development remains to be determined.

### 2.5. Additional Methylation Marks

In addition to the methylation types above, H3K79 and H4K20 specific lysine methylases could play a role during heart development.

**DOT1L** (HGNC alias: KMT4) catalyzes methylation at H3K79. *DOT1L* expression was downregulated in myocardial samples from patients with idiopathic dilated cardiomyopathy [115].

Cardiac-specific knockdown of *Dot1L* in mice resulted in chamber dilation, increased cardiomyocyte cell death, systolic dysfunction, and conduction abnormalities, as well as increased mortality rate. This phenotype is reminiscent of that in patients with dilated cardiomyopathy [115]. In the mouse cardiomyocytes, Dot1L acts through regulating the transcription of *Dystrophin* (*Dmd*), which affects the stability of the Dmd-glycoprotein complex that is required for cardiomyocyte viability [115]. Further, mouse cardiomyocyte models, in vitro and in vivo, showed that Dot1L drives a transitional pattern of H3K79me2 that is required for cardiomyogenesis [116].

**PRDM6** (HGNC alias: KMT8C) is involved in H4K20 methylation. A genetic variant in *PRDM6* has been associated with QRS duration (a measure of the conduction time from the atrioventricular node to the His-Purkinje system and ventricular myocardium). Changes in the QRS interval have been linked to disease progression for several cardiac etiologies, as well as to sudden death [117].

In mouse embryos, *Prdm6* is expressed in tissues known to be enriched in vascular precursor cells, including the heart, as well as in embryonic and adult-derived endothelial cell lines [118]. Overexpression of *Prdm6* induced apoptosis and inhibited proliferation in endothelial cells [118], indirectly suggesting a role in heart development.

Specific knockdown of *Prdm6* in mouse cardiac neural crest cells demonstrated its importance in embryonic heart development, as the hearts of deficient mice showed biventricular noncompaction and an inability to close the ductus arteriosus (small artery that connects the aorta and pulmonary artery) [119]; these findings were associated with reduced H4K20me1. Prdm6 was shown to regulate a network of genes required for cardiac neural crest specification, including *Wnt1*, *Tfap2b*, and *Sox9* [119].

Currently no evidence points towards a role for H4K20 methylation or any of its key components—KMT5A (HGNC alias: SETD8), KMT5B, and KMT5C—during heart development.

**Table 1:** **Variants in lysine-specific methyl transferases associated with heart disease.** KMT, lysine-specific methyltransferase; fs, frame-stop; N/A, detailed variant information not available. *, translation stop codon. Footnote ^1^: The authors apologize for any relevant studies that might have been omitted. Footnote ^2^: Patient also carried a variant in histone demethylase KDM5A (R1467W). Footnote ^3^: Both genetic variants were synonymous, i.e., they did not lead to an amino acid change in the protein sequence.

KMT	Variant	Cardiac Phenotype	Reference(s) (Footnote ^1^)
KMT2A	N/A	Congenital heart disease; Structural abnormalities	Sheppard et al., 2021 [31]
KMT2D	12 variants	Congenital heart defects; Kabuki syndrome	Van Laarhoven et al., 2015 [33]
	19 variants	Congenital heart defects; Kabuki syndrome	Digilio et al., 2017 [35]
	1 variant	Congenital cardiovascular malformation; Left-sided lesions	Li et al., 2017 [36]
	L3542P; G3553V	Congenital heart disease	Cuvertino et al., 2020 [37]
	R2860H; T1710M; V1561G	Congenital heart disease; Heterotaxy	Liang et al., 2020 [38]
	7 variants	Cardiac left-sided lesions; Hypoplastic left heart syndrome	Sun et al., 2020 [39]
	G3465Dfs * 37	Congenital heart defect; Left heart hypoplasia	Luo et al., 2021 [40]
KMT2C	N/A	Dilated cardiomyopathy	Jiang et al., 2017 [44]
	Q4753L (footnote ^2^)	Congenital heart disease; Ventricular septal defect	Szot et al., 2018 [45]
SMYD1	S91S; S321S (footnote ^3^)	Hypertrophic cardiomyopathy	Abaci et al., 2010 [52]
EHMT1	10 variants	Heart defects, unspecified; Kleefstra syndrome	Willemsen et al., 2012 [68]
PRDM16	R525Pfs * 79; K702 *; N816S	Left ventricular noncompaction; Deletion 1p36 syndrome	Arndt et al., 2013 [75]
	E271K; P291L; L887P; V1101M	Dilated cardiomyopathy; Deletion 1p36 syndrome	Arndt et al., 2013 [75]
	S350fs * 48	Dilated cardiomyopathy, pediatric	Long et al., 2017 [77]
	Q353 *	Cardiomyopathy; Left ventricular noncompaction	Delplancq et al., 2020 [76]
ASH1L	L1346 *	Anomalous coronary branching; Single left coronary	Homsy et al., 2015 [99]; Jin et al., 2017 [25]; Ji et al., 2020 [100]
	N/A	Congenital heart disease	Ji et al., 2020 [100]
NSD1	7 variants	Congenital heart defect, unspecified/Heart conduction defect	Cecconi et al., 2005 [108]
	A933P; R361S	Congenital heart disease; Atrioventricular septal defects	Priest et al., 2016 [110]
NSD2	N/A	Congenital heart disease	Ji et al., 2020 [100]
	10+ variants	Congenital heart defects; Wolf–Hirschhorn syndrome	Zanoni et al., 2021 [112]

## 3. Future Perspective

Genomic sequencing studies of cardiac disease, including those for patients with congenital heart disease, have identified large numbers of candidate disease genes [24,25,99]. Among them are many genes involved in histone modification (Table 1). While some of these have been validated in animal models [24,32], the contributions of most of these genes to congenital heart disease cannot be confirmed due to a lack of in vivo functional data in the context of heart development. Moreover, KMT catalytic activity is often induced by complex formation; therefore, the importance of cofactors and interactors of the KMTs in regulating heart development cannot be overstated, nor the important counterbalance and dynamics offered by KMDs. The rapid screening of large numbers of genes involved in histone modification necessitates a high-throughput model system, such as *Drosophila*, which has already demonstrated its potential in this regard [32].

Taken together, the existing literature indicate that each methylation of a histone-lysine, whether activating or repressing transcription, plays a role in heart development and, by extension, disease. But, whether it be their effect on cell fate decision and pattern specification during critical developmental stages or mediating cellular processes during development or in mature cells, methylation sites do not act in isolation. Ultimately, to comprehend the contribution of histone methylation in regulating the transcriptional transitions during processes of health and disease, we need to observe the entire methylation landscape. Initial studies towards this goal have already reported new insights. A study of multiple histone modifications (H3K4me3, active promoters; H3K27me3, inactive promoters; H3K4me1 and H3K27ac, promoters and enhancers), alongside the transcriptome at defined stages of cardiac differentiation (mouse embryonic stem cells to cardiomyocytes), identified complex, yet distinct, chromatin patterns that correlated with lineage decisions [120]. The data revealed distinct chromatin patterns that correlated to stage-specific gene expression, many of which were human candidate cardiac disease genes. They also revealed a multitude of chromatin patterns that rapidly diminished the expression of pluripotency-associated genes upon differentiation initiation, and chromatin patterns that could predict sets of functional related genes to orchestrate shared (early on) and distinguished (later stage) gene expression patterns during cardiomyocyte differentiation [120]. Another report studied H3K4me3 (transcription initiation), H3K36me3 (transcription elongation), and H3K27me3 (transcription repression) at five stages during the differentiation of human embryonic stem cells into cardiomyocytes [121]. It found that H3K4me2 and H3K27me3 temporal patterns during cardiomyocyte differentiation were more complex than the hitherto bivalent model, and demonstrated intricate histone-lysine pattern changes on the promotors of transcription factors known to have critical roles during cardiovascular development [121]. These studies set the stage for capturing the ever-increasing complexity of the changing methylation landscape and how it, as a whole, regulates the required intricacies for successful development of cardiac tissue.

The identification of methylation-associated genes in cardiac disease opens the door to new approaches in modifying disease-associated gene expression, such as administering small molecules that target specific KMTs. Pinometostat (EPZ-5676; Epizyme, Inc., Cambridge, MA, USA) is a DOT1L inhibitor and the first KMT inhibitor to enter a phase 1 clinical trial to study its safety and tolerability in patients with leukemia (NCT01684150) and a later phase 1/2 clinical trial (NCT03701295) to test its efficacy as a treatment for leukemia [122,123]. Since then, EZH2 inhibitors GSK2816126 (GlaxoSmithKline) and tazemetostat (EPZ-6438; Epizyme, Inc., Cambridge, MA, USA) have entered phase 1/2 clinical trials for the treatment of B-cell lymphoma (NCT02082977 and NCT01897571, respectively) [123,124]. Positive trial findings on safety and administration would encourage testing of these specific KMT inhibitors for their efficacy in treating congenital and other heart diseases associated with KMT variants. Like the genetic functional screens, the *Drosophila* models could be used for rapid, large-scale screens of candidate compounds [125,126,127,128]. Findings from the pharmacological screens (e.g., on efficacy and toxicity) in fly models could inform the prioritization of candidate compounds for subsequent testing in mammalian models, which are more costly and time-consuming, before ultimately moving to clinical trials.

## Data Availability

Not applicable.
